# Cyclophilin A‐mediated mitigation of coronavirus SARS‐CoV‐2

**DOI:** 10.1002/btm2.10436

**Published:** 2022-10-27

**Authors:** Simranjeet Singh Sekhon, Woo‐Ri Shin, Sang Yong Kim, Dong‐Seok Jeong, Wooil Choi, Bong‐Keun Choi, Jiho Min, Ji‐Young Ahn, Yang‐Hoon Kim

**Affiliations:** ^1^ Department of Microbiology Chungbuk National University Seowon‐Gu Cheongju South Korea; ^2^ Department of Food Science and Biotechnology Shin Ansan University Danwon‐Gu, Ansan Republic of Korea; ^3^ SEJONGBIO Heungdeok‐gu, Cheongju‐si Chungcheongbuk‐do Republic of Korea; ^4^ Graduate School of Semiconductor and Chemical Engineering Jeonbuk National University Jeonju Korea; ^5^ NUON Co., Ltd, Jungwon‐gu Seongnam Gyunggi Korea

**Keywords:** cyclophilin, COVID‐19 variants, coronavirus, spike protein receptor‐binding domain, surface plasmon resonance

## Abstract

Human cyclophilin A (hCypA) is important for the replication of multiple coronaviruses (CoVs), and cyclosporine A inhibitors can suppress CoVs. The emergence of rapidly spreading severe acute respiratory syndrome coronavirus 2 (SARS‐CoV‐2) variants has sparked concerns that mutations affect the binding ability of the spike (S) protein to the angiotensin‐converting enzyme 2 (ACE2) cell receptor, affecting the severity of coronavirus disease (COVID‐19). Far‐western blotting and surface plasmon resonance (SPR) results revealed that hCypA interacts strongly with the viral SARS‐CoV‐2 receptor‐binding domain (RBD), with a binding affinity of 6.85 × 10^−8^ M. The molecular interaction between hCypA and the viral protein interface was shown using three‐dimensional structural analysis, which revealed the blocking of key residues on the RBD interface by hCypA. The RBD facilitates binding to the ACE2 receptor. The hCypA–S protein complex suppressed the binding of RBD to the ACE2 receptor, which a required event for CoV entry into the host cell. The reliability of this postulated blocking mechanism of the hCypA–SARS‐CoV2 RBD complex with ACE was confirmed by SPR and molecular interaction lateral flow (MILF) strip assay, which offers the immunochromatographic signal read‐outs. The emergence of new SARS‐CoV‐2 variants with key mutations in RBD had a negligible effect on the binding of the RBD variants to hCypA, indicating an effective mitigation strategy for SARS‐CoV‐2 variants. The MILF strip assay results also highlight the neutralizing effect of hCypA by effectively blocking RBD (wild type and its variants) from binding ACE2. Given the importance of hCypA in viral entry regulation, it has the potential to be used as a target for antiviral therapy.

## 1 INTRODUCTION

An outbreak of severe acute respiratory syndrome coronavirus 2 (SARS‐CoV‐2) infection in December 2019 in Wuhan, China resulted in the new coronavirus (CoV) disease (COVID‐19), triggering a major public health concern worldwide.^1^ The World Health Organization (WHO) recognized COVID‐19 as a pandemic on March 11, 2020, and the number of infected cases has increased at an alarming rate worldwide. Specific anti‐CoV therapies and strategies are critical for the prevention and treatment of COVID‐19. Seven different CoVs (SARS‐CoV, hCoV‐NL63, hCoV‐HKU‐1, hCoV‐OC43, hCoV‐229 E, MERS‐CoV, and SARS‐CoV‐2) are currently known to cause respiratory illnesses in humans.^2^, ^3^ As a response to the SARS‐CoV‐2 infection, Paxlovid (Pfizer) was granted emergency use authorization by the United States Food and Drug Administration (FDA).^4^, ^5^ It contains the antiviral Nirmatrelvir/ritonavir, which is blocking the activity of SARS‐CoV2 main protease (Mpro) and 3CL protease (3CLpro).^6^ However, it may have serious side effects and may sometimes be fatal.^7^ Also, the appearance of SARS‐CoV‐2 variants of concerns (VOC) has created an urgent need to develop antiviral agents, new drugs, and vaccines to prevent infection.^8^

Cyclophilin (Cyp) proteins play a key role during the lifecycle of viruses from different families, such as human immunodeficiency virus, hepatitis C virus, dengue virus, Japanese encephalitis virus, yellow fever virus, hepatitis B virus, cytomegalovirus, human papillomavirus, influenza A virus, and vesicular stomatitis virus,^9^, ^10^ and are also important in the lifecycle of various CoVs. The lifecycles of SARS‐CoV, human CoV 229 E (HCoV‐229 E) and NL‐63 (HCoV‐NL63), responsible for mild respiratory infections in humans, and feline infectious peritonitis coronavirus (FPIV), responsible for a fatal disease in cats, are reported to be highly dependent on CypA.^11^, ^12^, ^13^, ^14^ Among the different Cyps, CypA is an important protein required for CoV replication and its inhibitor, cyclosporine A (CsA), has the ability to suppress CoV over a broad spectrum.^15^, ^16^, ^17^ The 18 kDa human cyclophilin A (hCypA) is an omnipresent protein belonging to the immunophilin family and is conserved and present in both eukaryotes and prokaryotes. hCypA has peptidyl‐prolyl *cis*‐*trans* isomerase (PPIase) activity that catalyzes the *cis*‐*trans* isomerization of peptide bonds at proline residues and regulates protein folding and trafficking.^10^

The CsA molecule can be used to inhibit the binding of hCypA to the SARS‐CoV‐2 receptor‐binding domain (RBD) and to control the hCypA mechanism.^18^, ^19^ In addition, the well‐known CsA molecule inhibits replication of various viruses by binding to intracellular human cyclophilins, which bind to the SARS‐CoV nucleocapsid protein.^16^, ^18^ CsA is an important immunosuppressive drug that inhibits PPIase activity by binding to both extracellular and intracellular CypA.^20^ It specifically inhibits the protein phosphatase calcineurin (Cn) and prevents the translocation of nuclear factor in activated T cells (NF‐AT) from the cytosol to the nucleus, thereby preventing the transcription of pro‐inflammatory cytokine encoding genes.^21^ While most studies have focused on the intracellular activities of cyclophilins, such as protein folding and molecular chaperone function,^22^ studies on the extracellular activity of CypA are limited.

SARS‐CoV and MERS‐CoV virions carry sufficient quantities of CypA to maintain their lifecycle and facilitate defects in cell production in their target cells.^15^ CypA has also been reported to interact intracellularly with nonstructural SARS‐CoV protein 1 (Nsp1).^12^ The N protein of SARS‐CoV also binds closely to hCypA, and the protein complex formation can be inhibited by CsA, blocking viral replication.^10^, ^14^, ^23^ In this context, it is not surprising that CsA, which is a potent hCyp inhibitor with immunosuppressive anti‐calcineurin properties, inhibits the in vitro replication of various CoVs, such as HCoV‐229 E, HCoV‐NL63, FPIV, mouse hepatitis virus (MHV), avian infectious bronchitis virus (IBV), and SARS‐CoV, which are genetically close to SARS‐CoV‐2.^17^, ^23^, ^24^

The homotrimeric spike (S) glycoprotein mediates SARS‐CoV‐2 entry through the angiotensin‐converting enzyme 2 (ACE2) receptor on the host cell membrane.^25^ ACE2 receptor recognition by SARS‐CoV‐2 in humans is similar to that observed in the 2003 SARS‐CoV. The human receptor ACE2 is expressed as a membrane‐bound protein present in various organs.^26^ At the initial stage of viral replication, binding to the ACE2 receptor is crucial for the entry of SARS‐CoV‐2 into target cells.

The RBD of S1 includes a core and a receptor‐binding motif (RBM), with residues 438–506, that specifically recognizes ACE2.^26^ Leu455, Phe456, Ser459, Gln474, Ala475, Phe486, Phe490, Gln493, Pro499, and Asn501 are the key residues in the RBM of the SARS‐CoV‐2S protein that facilitates the binding of the ACE2 receptor, as has been revealed from the cryo‐EM structures of the SARS‐CoV‐2–hACE2 complex.^27^, ^28^, ^29^ RBD is crucial for determining cross‐species and human‐to‐human transmissibility.^28^ Antibodies have been used to bind the SARS‐CoV‐2 RBM in numerous studies, it can be a neutralizer of SARS‐CoV‐2 and generate information on the nature of immune responses.^30^, ^31^, ^32^ To gain insights into the function of hCypA in the SARS‐CoV‐2 life cycle, we identified and analyzed the interactions of hCypA with S proteins of SARS‐CoV‐2. These findings also highlight the unique structural characteristics of SARS‐CoV‐2 RBD, which will help us understand the molecular mechanisms of viral infection.

The emergence of rapidly spreading SARS‐CoV‐2 variants has sparked concerns about reduced vaccine efficacy. Researchers have found that variants with mutations, which have significant biological functions, have high transmissibility, indicating that key mutations may affect the severity of COVID‐19 and viral spread and prevent natural or vaccine‐induced immunity. These key mutations significantly affect the binding ability of the S protein to the ACE2 receptor. In this study, we also analyzed the molecular interactions of the SARS‐CoV‐2 variants with the hCypA protein to determine the effect of variants on the binding and blocking potential of the hCypA–S protein complex with the ACE2 receptor. In the present study, we affirmed the hCypA interaction with RBD and the interference in binding ACE2‐RBD for SARS‐CoV‐2. A molecular interaction lateral flow (MILF) strip assay was also constructed to determine the inhibitory effect of hCypA on SARS‐CoV‐2 RBD and its variants. Finally, the hCypA protein–RBD complex suppressed the binding of RBD to the ACE2 receptor, which was a required event for SARS‐CoV‐2 entry into the host cell. This study provides an important opportunity to determine the efficacy of hCypA as a potential treatment drug target for COVID‐19.

## 2 RESULTS

### 2.1 hCypA S protein RBD interactions

**FIGURE 1:**
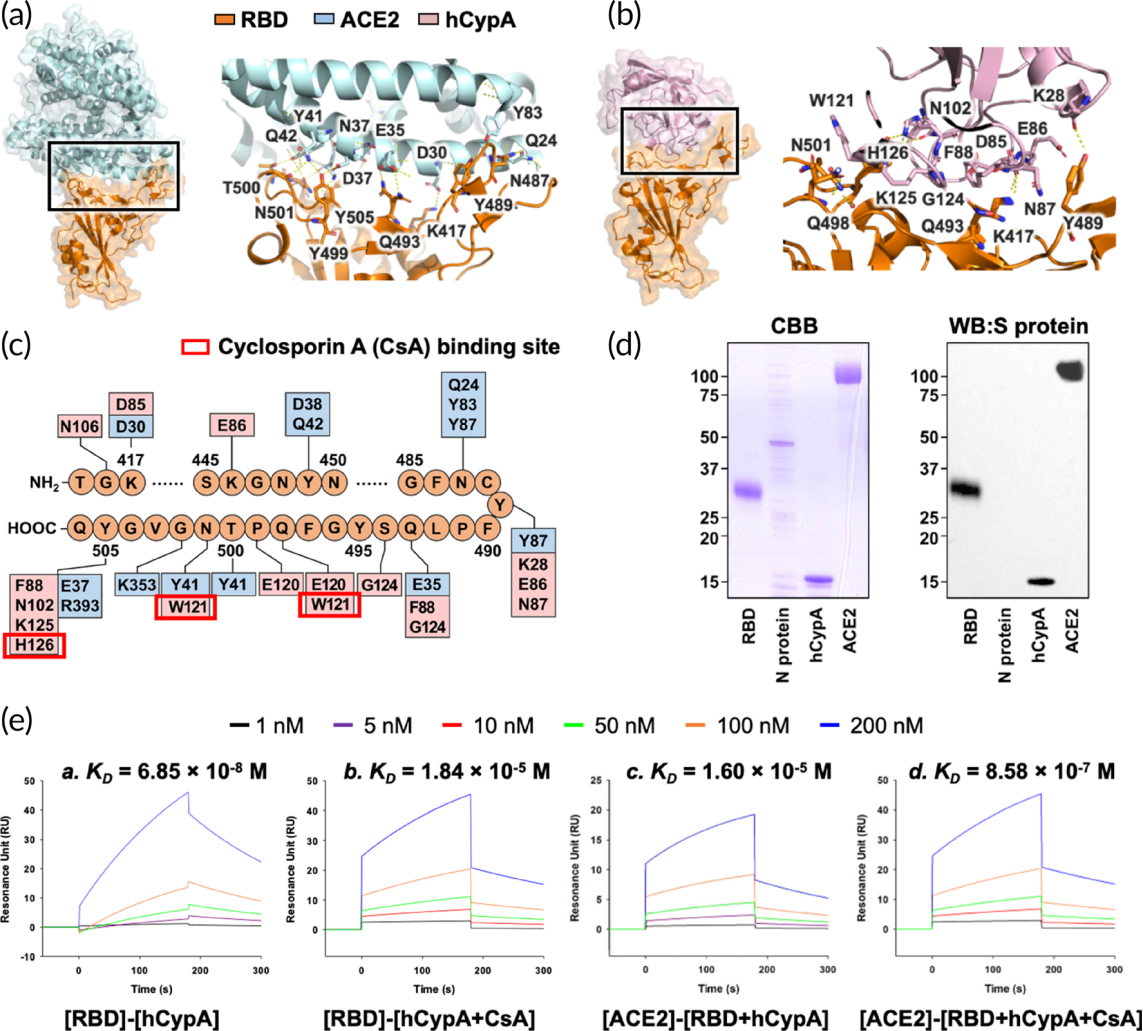
(a) Structural representation of the S protein RBD (orange) complexed with SARS‐CoV‐2 receptor ACE2 (light blue). The key residues that take part in the interaction are also shown. (b) Structural representation of hCypA (pink) and S protein RBD (orange) docked complex with interacting region residues labeled on the complex structure. (c) The integrated interaction map of S protein RBD–ACE2 complex and S protein RBD–hCypA complex, highlighting the overlapping regions and residues on RBD. (d) Far‐western blotting results showing hCypA S protein RBD interactions. CBB, Coomassie Brilliant Blue staining; WB:S protein, Western Blotting signaled with anti‐spike protein antibody. (e) The SPR binding affinity of [RBD] binding to [hCypA] (a), [RBD] to [hCypA + CsA] (b), [ACE2] to [RBD + hCypA] (c) and [ACE2] interaction with [RBD + hCypA + CsA] complex (d) are shown.

The SARS‐CoV‐2S protein is very similar in sequence (80% sequence identity) and structure (RMSD = 0.411 Å) to the S protein of SARS‐CoV (Figures 1a and S1A–C). The CoV intervention strategies aimed at blocking the receptor recognition of SARS‐CoV‐2S protein can be very useful in restricting the interaction of SARS‐CoV‐2 with ACE2, thereby preventing virus entry into the target cells. As shown in Fig. S1Ea, the surface plasmon resonance (SPR) binding affinity *K*_*D*_ value of 4.48 × 10^−8^ M (48.8 nM) indicates that ACE2 binds marginally strongly to the SARS‐CoV‐2 S protein relative to SARS‐CoV.^27^ The binding affinities between ACE2 and SARS‐CoV RBDs is the similar binding affinity (*K*_*D*_ value of 1.0 × 10^−8^ to 6.0 × 10^−8^ M [10–60 nM]) based on the reported binding results.^33^, ^34^ (Figure S1G). Structural analysis of SARS‐CoV‐2 RBD showed that the ACE2 binding mode was almost identical and that most binding residues conserved or shared similar side chain properties (Figures 1a and S1F). Structural analysis provides a precise target for the binding of hCypA to the SARS‐CoV‐2 RBD (Figure 1b). To compare the ACE2‐interacting residues on the RBD with the hCypA–RBD complex, we employed a structure‐guided interaction mapping approach to determine similarities in the binding region (Figure 1c). The hCypA protein engulfs the RBM of SARS‐CoV‐2 and blocks access to key residues Leu455, Phe456, Ser459, Gln493, Pro499, and Asn501, which participate in the interaction with ACE2. The overall binding interface of RBD to hCypA was similar to that observed in the RBD–ACE2 complex. It is the loop region on the hCypA surface that covered the RBM residues. The polar residues of the hCypA side chain Glu86 and Asn87 form hydrophobic interactions with key RBM hydrophobic residues Leu455 and Phe456 and block their surface. Gln493 in the RBM is also obstructed by the flexible loop residue Phe88 of hCypA. The hCypA active site residues directly participate in interactions with the RBM S protein. The hCypA's active site groove consists of seven residues (His54, Arg55, Phe60, Gln111, Phe113, Trp121, and His126) that take part in the PPIase activity, and interactions with CsA (Figure S1B,Ec). Trp121 and His126 present on the side of the cleft opening bind to RBD and partially block access to the hCypA active site. The His126 residue forms a hydrogen bond with the Tyr505 residue of RBM, 2.11 Å apart. The Trp121 residue has strong hydrophobic interactions with Asn501 and completely covers the residue interface. Asn501 is an important residue that binds Tyr41 of ACE2 and forms a hydrogen bond. The Gly124 loop residue on hCypA also forms a hydrogen bond with Tyr453 on the RBM, separated by 1.95 Å. Gly124 also surrounds Gln493 with strong hydrophobic interactions and binds to the RBM region (Figure 1c). The hCypA–RBD complex interface was stabilized by numerous interactions and a series of five apparent intermolecular hydrogen bonds (Table 1).

**TABLE 1:** Residue interactions of S protein RBD–ACE2 complex and S protein RBD‐hCypA complex

SARS‐CoV‐2 RBD	ACE2	Length of hydrogen bond (Å)	hCypA	Length of hydrogen bond (Å)
Y505	R393	3.40	N102	3.02
	G354		K125	
	E37	2.60	H126	2.11
G502	K353	2.78		
**N501**	K353		W121	
	Y41			
T500	D355			
	N350			
	Y41	2.71		
	R357			
Q498	Q42		E120	
	Y41		W121	
G496	K353			
S494			G124	
**Q493**	E35	2.69	G124	
			F88	
Y489	T27		N87	
	F28		E86	
	Y83		K28	3.10
N487	Y83			
	Q24	2.69		
**F486**	L79			
	M82			
	Y83			
E484			I89	
**F456**	T27		E86	
			N87	
**L455**	H34		N87	
Y453	H34		F88	
			G124	3.00
Y449	D38	2.70		
	Q42	2.79		
G446	Q42	3.24		
K417	D30	2.90	D85	3.24
G416			N106	
Q409			P105	
E406			P105	
R403			K125	
			G104	
			P105	

*Note*: Bold: Neutralizing key amino acids (L455, F456, S459, Q474, A475, F486, F490, Q493, P499, and N501).

To gain insights into the interaction between SARS‐CoV‐2 RBD and hCypA, protein–protein interaction analysis was performed. Far‐western blotting detects a target “prey” protein on the membrane using antibody‐detectable “bait” protein.^35^ Far‐western blotting results confirmed the binding of RBD to hCypA and the ACE2 receptor (Figure 1d). Each protein was loaded on SDS‐PAGE, and the RBD was detected using an RBD antibody. The RBD band (control) and ACE2 band signal appeared clearly, indicating that RBD interacts with ACE2 in the PVDF membrane. In addition, the hCypA band signal appears to be smaller than 15 kDa (hCypA protein size 13 kDa), indicating that hCypA interacts with the RBD. Moreover, the SPR results showed that hCypA binds to SARS‐CoV‐2 RBD with a binding affinity *K*_*D*_ value of 6.85 × 10^−8^ M (68.5 nM) and binding energy of −65.0146 kcal mol^−1^ (Figure 1e,a). The stability and strong interaction between the two proteins (hCypA and SARS‐CoV‐2 RBD) are evident from their binding affinity, as well as from the key amino acid interactions on RBM. The binding affinity of ACE2 receptor with RBD significantly reduces in the presence of hCypA with *K*_*D*_ of 1.60 × 10^−5^ M for [ACE2]‐[RBD + hCypA], as compared to *K*_*D*_ value 4.48 × 10^−8^ M (44.8 nM) for [ACE2]‐[RBD] (Figures 1e,c and S1Ea). hCypA does not prefer binding to the ACE2 receptor, and a low affinity *K*_*D*_ value of 1.17 × 10^−4^ M affirms this finding (Figure S1Eb). CsA is an immunosuppressive drug that prevents hCypA activity by binding tightly to the active site residues (Figure S1D,Ec). The SPR results showed that RBD interactions with the hCypA–CsA complex were fairly reduced with a low *K*_*D*_ value of 1.84 × 10^−5^ M in comparison to enzymatically active hCypA (Figure 1Eb). Notably, only a distinct binding conformation of hCypA was observed in the crystal structure of a complex of CsA.^36^ The active site groove takes part in the PPIase activity and interactions with CsA (Figure S1D). However, it is not clear how the compact conformation of hCypA–CsA affects the ACE2–RBD complex. Comparison of the structure analysis suggested that the amino acids Trp121, Gly124, and His126 (hCypA numbering) affect CsA interactions. These residues maintain interaction with Asn501, Gln493, and Tyr505 of SARS‐CoV‐2 RBD. CsA binds tightly to hCypA with *a K*_*D*_ value of 2.07 × 10^−10^ M (Figure S1Ec). The hCypA–CsA complex binding affinity (*K*_*D*_ value of 2.07 × 10^−10^ M) was higher than that of the hCypA–RBD complex binding affinity (*K*_*D*_ value of 6.85 × 10^−8^ M). When the RBD, hCypA, and CsA were all present, hCypA and CsA were more likely to form a complex. This may be because blocking of active site residues of hCypA with CsA weakens the interactions of hCypA with RBD. With the key residues on RBD open due to the binding of hCypA–CsA, ACE2 favorably binds the RBD complex with a *K*_*D*_ value of 8.58 × 10^−7^ M (Figure 1Ed). Additionally, CsA showed no binding affinity for either ACE2 or RBD (Figure S1Ee,d). These results suggest that extracellular hCypA can be used as an effective mitigating agent for SARS‐CoV‐2 by hindering the binding of RBD to the ACE2 receptor and blocking its entry into the cell.

### 2.2 hCypA and SARS‐CoV‐2 variants

To gain molecular insights into the structural difference of hCypA complexed with the SARS‐CoV‐2 variants, that is, Alpha (United Kingdom, B.1.1.7: N501Y), Beta (South Africa, B.1.351: K417N, E484K, N501Y), Gamma (Japan/Brazil, P.1: K417T, E484K, N501Y), and Delta (India, B.1.617.2: L452R, T478K), Omicron (South Africa BA.1(B.1.1.529): G339D, S371L, S373P, S375F, K417N, N440K, G446S, S477N, T478K, E484A, Q493R, G496S, Q498R, N501Y, Y505H) and additional variants of interest Epsilon (US‐California, B.1.427: L452R), Kappa (India, B.1.617.1: L452R, E484Q), and Lambda (Peru, C.37; L452R, F490S), structural analysis was performed on each RBD–hCypA complex (Table 2). Additionally, the Deltacron (AY.4/BA.1: G339D, S371L, S373P, S375F, K417N, N440K, G446S, L452R, S477N, T478K, E484A, Q493R, G496S, Q498R, N501Y, Y505H) was analyzed to observe the structural difference with hCypA.

**TABLE 2:** hCypA residue interactions with SARS‐CoV‐2 variants

hCypA	Wild type	Alpha	Beta	Gamma	Delta	Epsilon	Eta	Kappa	Lambda	Omicron	Deltacron
V12						Y505		R403 Y505	Y505		
D13						R403 K417 Y453		R403 K417 Y453 Y505	R403 K417 Y453		
G14						K417		K417	K417		
E15						R403		R403	R403		
P16										**F486**	**F486**
L17								Y505		**F486**	**F486**
G18										**F486**	**F486**
T41					R408						
D27	**F486**										
K28	Y489										
G42					R408						
K44		Q498		Q498	T376 K378						
G45		G446 Q498		G446 Q498	R408 A411	**F486**		**F486**	**F486**		
F46		G446Q498			W380	**F486**		**F486**	**F486**		
G47					Q414						
K49					R408 Q414 T415 G416						
G50					G413 T415	Y489		Y489	Y489		
H54			Y505								
R55			R403 Y505								
P58										Y449	Y449
G59										Y449 S496 R498	S496
F60			**L455**							R498	R498
F67		Y449		Y449	P412	**F486**		**F486**	**F486**		
T68		Y449		Y449		G485 **F486**		G485 **F486**	G485 **F486**		
R69				**L452** **Q493** S494	D427 D428						
H70					G413	E484 G485 C488 Y489		E484 G485 C488 Y489	E484 G485 C488 Y489		
N71			Y505								
G72		K484		K484							
T73		K484 **F490**	R408	K484 **F490**							
K76		Y449 G496 **Y501**		Y449 G496 **Y501**	C379 W380		F486				
I78							G485				
Y79							Y489				
G80							Y489				
E81		Y453		Y453 **L455** **Q493**			Y489				
K82		Y489		Y489							
E84							S494				
D85	K417						Y449				
E86	**F456** Y489						Y449				
N87	**L455** **F456** Y489										
F88	Y453 **Q493**						G446 Y449 Q498				
I89	E484										
T93										H505	H505
N102	Y505										
A103		G485 C488 Y489		G485 C488 Y489							
G104	R403	**F486** N487	T415	**F486** N487							
P105	R403 E406 Q409	N487	T415	N487			Y505				
N106	G416						Y453 S494				
T107				Y489							
T116										**R493**	**R493**
A117			Y489							**Y501**	**Y501**
K118										G502 H505	G502 H505
E120	Q498									T500	T500
W121	Q498 **N501**		Y421 **F456** R457 Y473								
G124	Y453 **Q493** S494						Q498				
K125	R403 Y505	**F486**	D420	**F486**							
H126	Y505										
N137										**F456** Y489	**F456** Y489
I138										Y489	Y489
G140										**L455** **R493** P494	**L455** **R493** P494
F145								Y505			
R148			Q493								
N149			Y449 S494 G496 **Y501**								
K151			**Y501**			Y449		Y449	Y449		
S153						Y495 G496 Q498		Y495 G496 Q498	Y495 G496 Q498		
K154						G496 Y505		R403 G496 Y505	R403 G496 Y505		
K155						**Q493**		**Q493**	**Q493**		
D160					R408						
C161					R408						

*Note*: Bold: Neutralizing key amino acids (L455, F456, F486, F490, Q493, P499, and N501).

**FIGURE 2:**
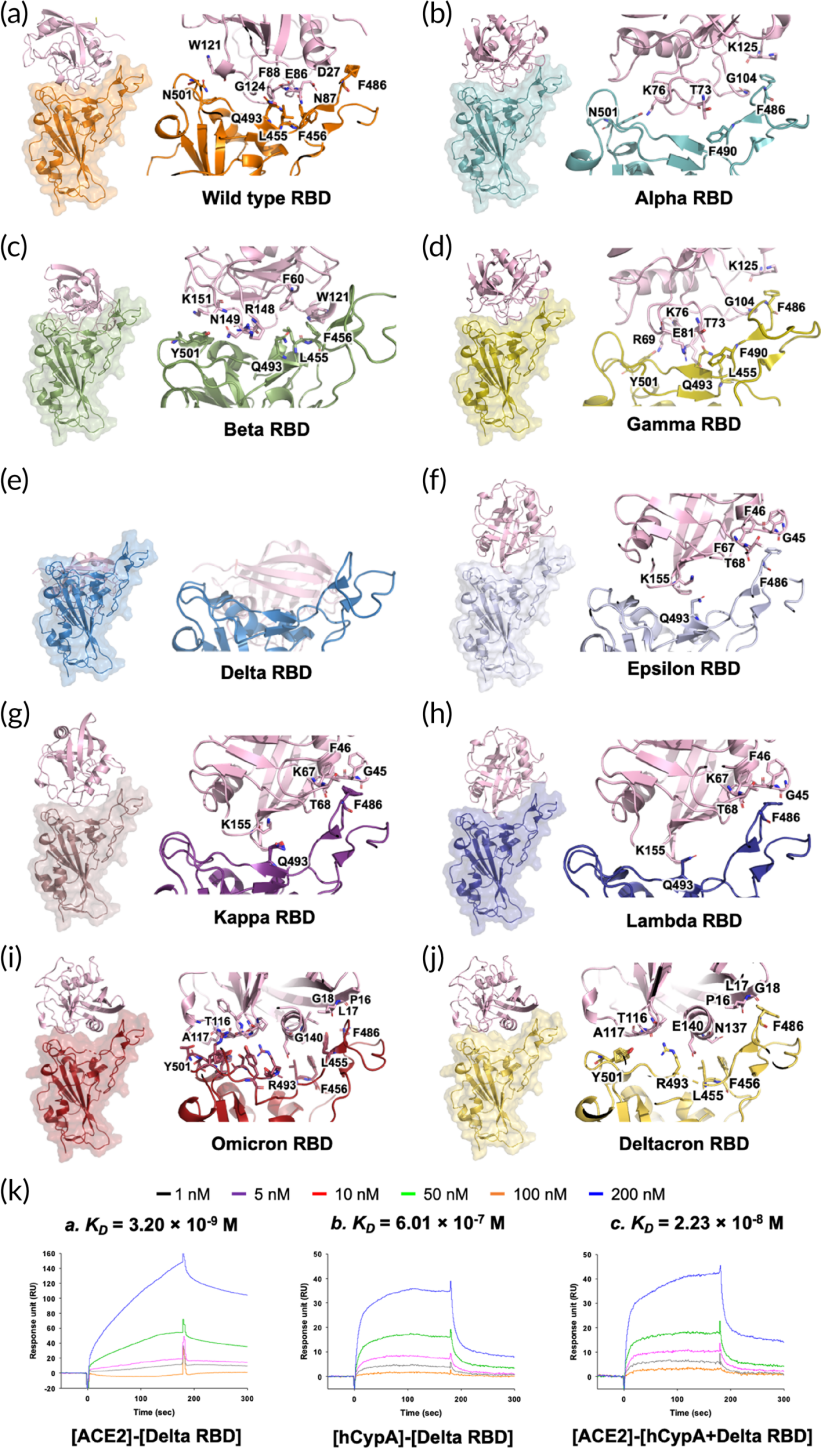
Structural representation of the S protein RBD variants complexed with hCypA (pink). (a) S protein RBD wild type (orange) and hCypA complex, (b) Alpha RBD and hCypA complex, (c) Beta RBD and hCypA complex, (d) Gamma and hCypA complex, (e) Delta RBD and hCypA complex, (f) Epsilon–hCypA complex, (g) Kappa RBD–hCypA complex, (h) Lambda–hCypA complex, (i) Omicron–hCypA complex, and (j) Deltacron–hCypA complex. (k) SPR affinity analysis of the [ACE2]‐[Delta RBD] is (a), [hCypA]‐[Delta RBD] is (b), and [ACE2]‐[hCypA + Delta RBD complexes] is (c).

**FIGURE 3:**
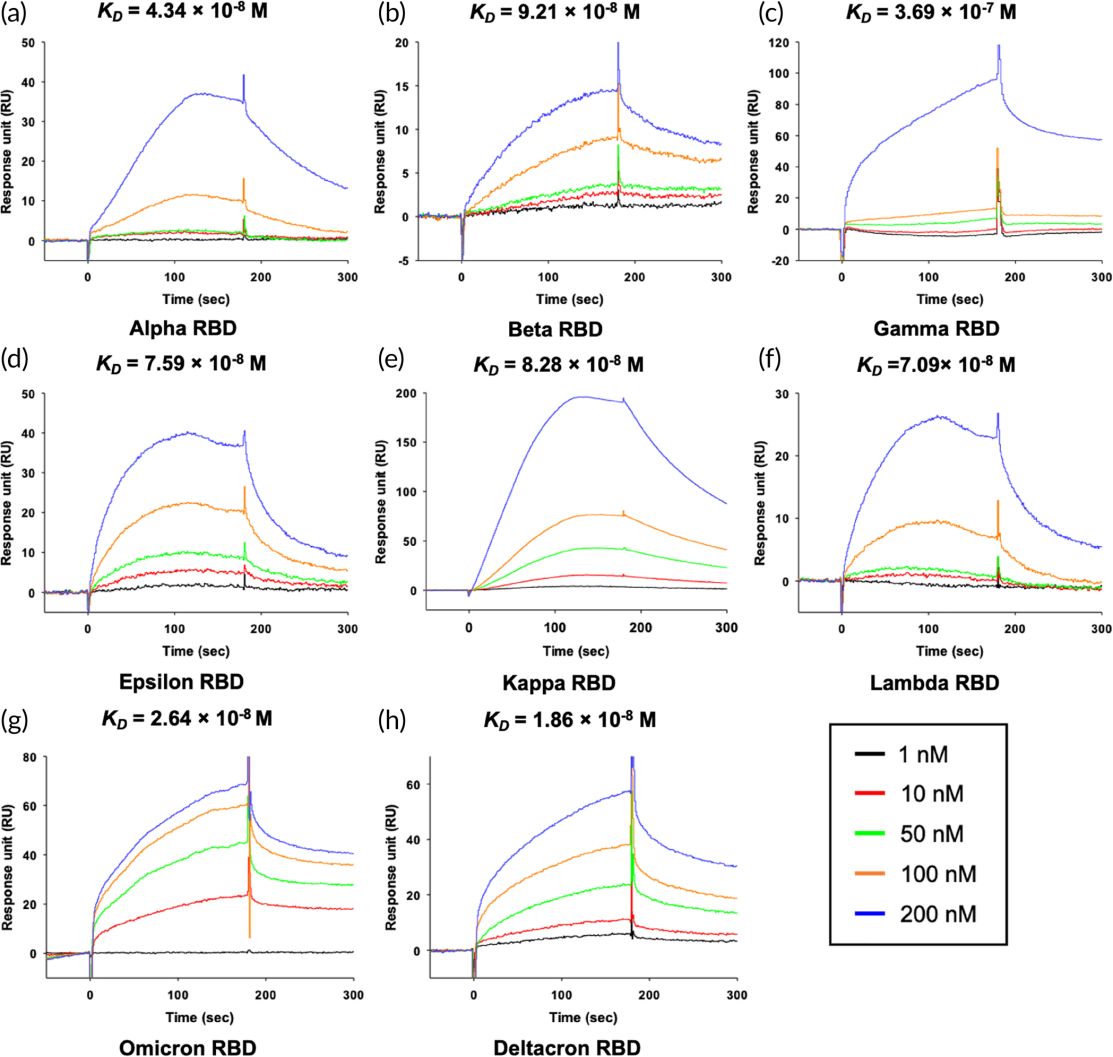
SPR affinity analysis of the hCypA‐RBD variants. (a) [hCypA]‐[Alpha RBD], (b) [hCypA]‐[Beta RBD], (c) [hCypA]‐[Gamma RBD], (d) [hCypA]‐[Epsilon RBD], (e) [hCypA]‐[Kappa RBD], (f) [hCypA]‐[Lambda RBD], (g) [hCypA]‐[Omicron RBD], and (h) [hCypA]‐[Deltacron RBD]. The *K*_*D*_ value of binding affinity is shown in Table 3.

The wild‐type RBD–hCypA complex (Figure 2a) was used as a reference to determine the structural changes caused by mutations in the RBD of the variant. Structural analysis of nine variants (Alpha [Figure 2b], Beta [Figure 2c], Gamma [Figure 2d], Delta [Figure 2e], Epsilon [Figure 2f], Kappa [Figure 2g], Lambda [Figure 2h], Omicron [Figure 2i], and Deltacron [AY.4/BA.1] [Figure 2j]) was performed with hCypA, suggesting no significant structural alterations except Delta. The Phe486 and Tyr505 residues of RBD on variants that are critical for binding ACE2 also bound hCypA, suggesting an inhibitory effect aided by the hCypA‐variant RBD complex on ACE2. SPR analysis was performed to determine the binding ability of the variants to the ACE2 receptor. All seven variants were found to bind strongly to ACE2, with *K*_*D*_ values ranging between (10^−8^ to 10^−9^ M) (Figure 3 and Table 3). We emphasize that hCypA is able to bind to Alpha, Beta, Gamma, Epsilon, Kappa, Lambda, Omicron, and Deltacron (AY.4/BA.1) except Delta. The delta variant is more high risk of death than the other variants of concerns.^37^, ^38^, ^39^ The SPR result of the [ACE2]–[Delta RBD] complex showed strong binding with a *K*_*D*_ value of 3.20 × 10^−9^ M (Figure 2k,a). The T478K mutation in the flexible loop region with a long side chain increased stability during interaction with ACE2. The Delta RBD bound hCypA with a *K*_*D*_ value of 6.01 × 10^−7^ M (Figure 2k,b). It is postulated that the T478K mutation increases the electrostatic potential to the positive surface and causes steric hindrance during interaction with hCypA. The hCypA‐binding interface on delta RBD is completely different from the hCypA wild‐type RBD complex, with no key residue or RBM facilitating region binding to the ACE2 receptor, which either binds hCypA or is blocked by hCypA (Figure 2e). The open Delta RBM region corresponded to tight binding of ACE2 with the [hCypA + Delta RBD] complex with a *K*_*D*_ value of 2.23 × 10^−8^ M (Figure 2k,c). These results suggest that hCypA is unable to block the entry of the delta variant into the host cell. The remaining six variants bound to hCypA with high affinity (10^−7^ to 10^−8^ M) and overlap the ACE2 binding region on wild‐type RBD. The hCypA flexible loop region occupied the key residues in wild type RBD and prevented the protein from tightly binding to the ACE2 receptor. The hCypA plus variant RBD complexes inhibited the binding with ACE2, and *K*_*D*_ value range 10^−4^ to 10^−5^ M confirmed the blocking effect of hCypA on variants (Figure S2). hCypA strongly binds omicron variant RBD with binding energy similar to the wild type [RBD]–[hCypA] complex (Figure 1e). hCypA overlaps the ACE2 binding residues on omicron RBD and has direct hydrophobic interactions with Leu455, Phe456, and Phe486 key residues on RBD blocking ACE2 access. hCypA also interacts with five key mutated omicron RBD (Arg493, Ser496, Arg498, Tyr501, and His505) residues forming strong hydrogen bonds with Arg493 and His505 residues. The inhibition of RBM on variant S proteins by hCypA affirmed its potential role as COVID‐19 mitigating agent.

**TABLE 3:** SPR binding affinity values of ACE2–RBD variants, hCypA–RBD variants, and ACE2 with hCypA–variants RBD complex

Ligand	Receptor (SARS‐CoV‐2 RBD)	Ka (1/M*s)	Kd (1/s)	*K*_*D*_ (M)
ACE2	Alpha	3.08 × 10^4^	2.14 × 10^−4^	6.97 × 10^−9^
	Beta	1.02 × 10^6^	2.887 × 10^−2^	2.83 × 10^−8^
	Gamma	2.88 × 10^5^	1.002 × 10^−3^	3.49 × 10^−9^
	Delta	2.57 × 10^5^	8.21 × 10^−4^	3.20 × 10^−9^
	Epsilon	2.55 × 10^6^	2.153 × 10^−1^	8.46 × 10^−8^
	Kappa	9.76 × 10^4^	3.682 × 10^−3^	3.77 × 10^−8^
	Lambda	2.50 × 10^5^	1.753 × 10^−3^	7.01 × 10^−9^
	Omicron	2.58 × 10^4^	2.293 × 10^−3^	8.90 × 10^−8^
	Deltacron	6.59 × 10^4^	4.519 × 10^−4^	6.85 × 10^−9^
hCypA	Alpha	2.37 × 10^5^	1.029 × 10^−2^	4.34 × 10^−8^
	Beta	4.04 × 10^5^	3.719 × 10^−2^	9.21 × 10^−8^
	Gamma	2.25 × 10^4^	8.305 × 10^−3^	3.69 × 10^−7^
	Delta	6.60 × 10^4^	3.97 × 10^−2^	6.01 × 10^−7^
	Epsilon	3.17 × 10^4^	2.405 × 10^−3^	7.59 × 10^−8^
	Kappa	1.14 × 10^5^	9.444 × 10^−3^	8.28 × 10^−8^
	Lambda	5.38 × 10^4^	3.815 × 10^−3^	7.09 × 10^−8^
	Omicron	6.26 × 10^4^	1.643 × 10^−3^	2.64 × 10^−8^
	Deltacron	5.77 × 10^4^	1.072 × 10^−3^	1.86 × 10^−8^
ACE2	hCypA + Alpha complex	2.88 × 10^2^	3.788 × 10^−3^	1.32 × 10^−5^
	hCypA + Beta complex	7.63 × 10^2^	5.889 × 10^−2^	7.72 × 10^−5^
	hCypA + Gamma complex	6.51 × 10^2^	4.879 × 10^−2^	7.49 × 10^−5^
	hCypA + Delta complex	1.39 × 10^4^	3.10 × 10^−4^	2.23 × 10^−8^
	hCypA + Epsilon complex	2.48 × 10^0^	1.92 × 10^−4^	7.74 × 10^−5^
	hCypA + Kappa complex	1.23 × 10^0^	2.30 × 10^−4^	1.87 × 10^−4^
	hCypA + Lambda complex	1.76 × 10^0^	3.55 × 10^−4^	2.02 × 10^−4^
	hCypA + Omicron complex	1.44 × 10^0^	2.98 × 10^−4^	2.08 × 10^−4^
	hCypA + Deltacron complex	1.69 × 10^0^	2.46 × 10^−4^	1.46 × 10^−4^

*Note*: The graph of SPR affinity analysis is shown in Figure 3 and Figure S2.

### 2.3 MILF strip assay

**FIGURE 4:**
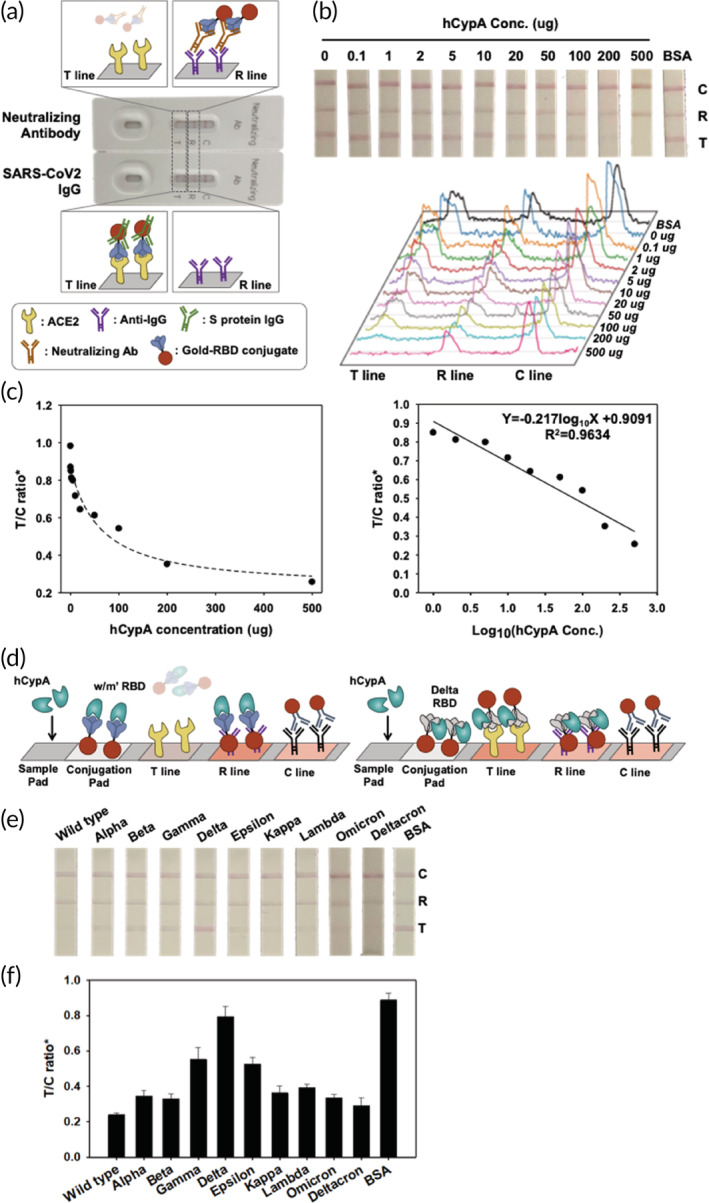
(a) Illustration of the MILF strip assay for neutralizing antibody test to SARS‐CoV‐2 and evaluation of the analytical performance of the SARS‐CoV‐2 MILF strip. (b) Binding interference between ACE2 and SARS‐CoV‐2 RBD was tested using hCypA. The images and signals of strips exposed to different hCypA concentrations were analyzed by ImageJ software. (c) The calibration graph as control line (C line) and test line (T line, ACE2 zone) ratio of hCypA concentration. (d) MILF strip assay that highlights the neutralizing ability of hCypA was confirmed at the T line using AuNP‐variant RBDs. MILF strip assay test results of the hCypA with the AuNP‐variants RBDs (e), and the calibration graph as the T/C ratio (f).

**FIGURE 5:**
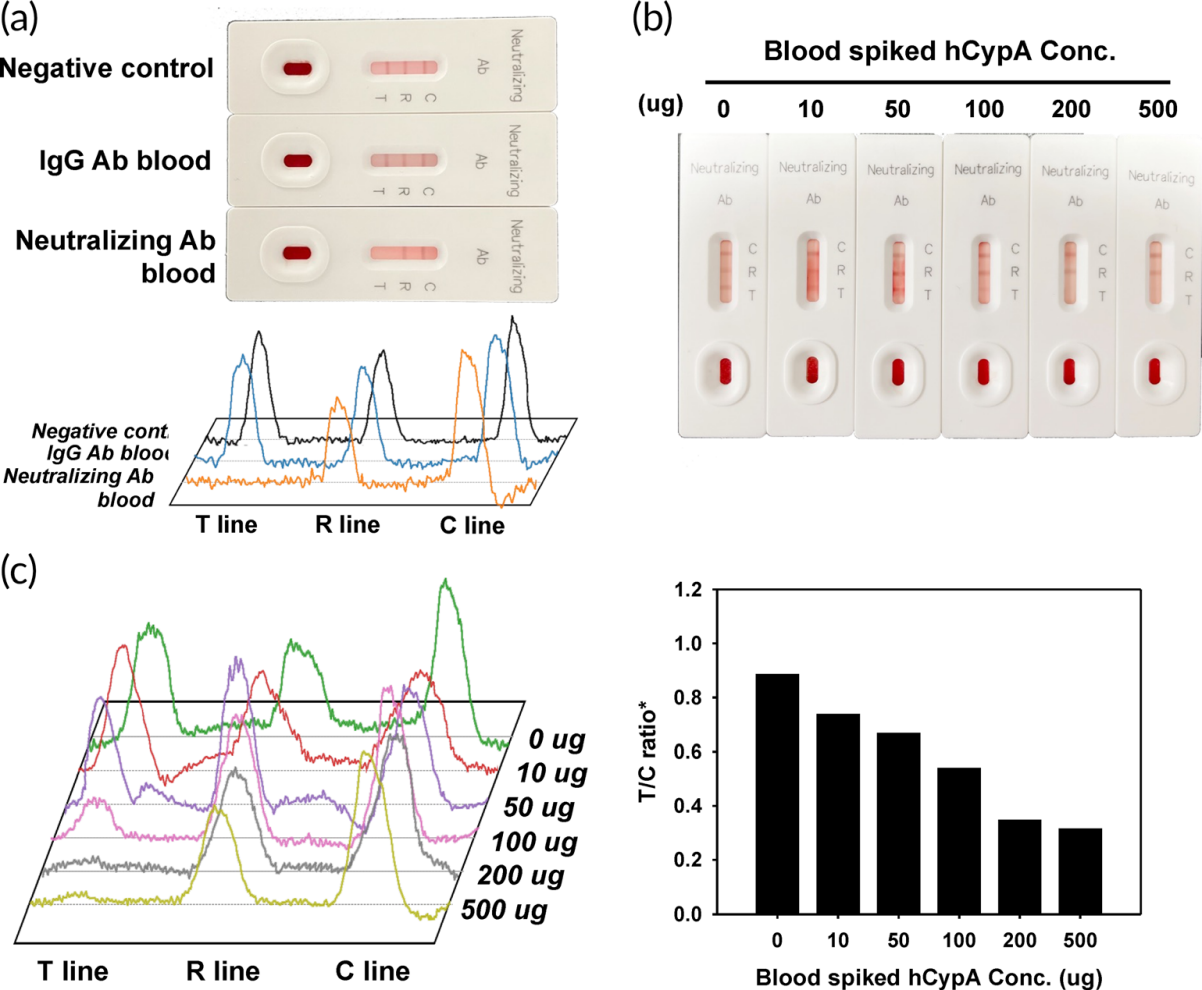
(a) Image of the MILF strip assay for neutralizing antibodies against SARS‐CoV‐2 using standard blood samples. (b) Response of the MILF strip in hCypA‐spiked human blood (without IgG and neutralizing antibodies). (c) Band signal intensity and calibration graph of hCypA‐spiked human blood.

Based on the above analysis, we designed a MILF strip assay using SARS‐CoV‐2 RBD. MILF strip assay determined the molecular interaction of hCypA on SARS‐CoV‐2 RBDs by developing a competitive method. We sequentially optimized MILF strips with different amounts of RBD IgG and neutralizing antibody deposited on the sample pad, that is, 1 μg/mm^2^. As the MILF strip assay is generally based on visual observation, gold nanoparticles are ideal and stable materials for color. The neutralizing antibody reacts to the ACE2 binding site in the RBD to inhibit AuNP‐RBD on the conjugation pad from binding to ACE2 at the T line. Because IgG binds to the RBD variably, the AuNP‐RBD–IgG complex can bind to ACE2 on the T line (Figure 4a). Analytical evaluation was then performed using hCypA samples. Figure 4b shows images of the MILF strip bands exposed to samples containing different concentrations of hCypA. This shows that the band intensity disappears at the T line (ACE2 zone) as the hCypA concentration increases. It showed that the strips deposited with 0.01 μg of hCypA had 1.5 times the band intensity than those with 200 μg of hCypA. Regression analysis was performed based on the feature parameters (T/C ratio) (Figure 4c). The ratio of the T and C line is the most important parameter that affects the efficiency of target recognition and the signal ability. The ratio of R/C represents the control value as in the C line because Anti‐IgG is fixed on the R line. The R/C ratio is not changed despite the various concentrations of hCypA (Figure S3A,B). The T/C ratio observed over Log_10_(hCypA Conc.) displayed a linear graph, confirming that hCypA inhibited the binding of RBD to ACE2. To determine whether hCypA can inhibit other variants of RBD, we performed a MILF strip assay analysis with the AuNP‐variant RBD complex on the conjugation pad. The MILF strip assay with all AuNP‐variants' RBD (except Delta RBD) resulted in the band disappearing on the T line, as the hCypA overlaps at the same binding site as ACE2. For the AuNP‐Delta RBD immobilized on the MILF strip, hCypA bound to an alternate flexible loop region on RBD, and the presence of the T line band confirmed this result (Figure 4d). The results of the reaction of 200 μg hCypA in the strip sensor, immobilized with AuNP‐variant RBD on the conjugation pad, showed that a similar band appeared on the T line of the AuNP‐delta RBD MILF strip, as also indicated by the BSA control (Figure 4e). Moreover, the band intensity of the T line between wild‐type RBD and RBD variants was compared. As shown in Figure 4f, the band intensity of Delta RBD was higher than that of the other RBD variants and four times stronger than that of the wild‐type RBD. The gamma and epsilon variants also showed a weak band intensity and were significantly lower than that of the delta variant. We further verified the activity of SARS‐CoV‐2 neutralizing antibody with MILF strip assay, FIA, and ELISA (Figure S3C–F). We then compared the extracellular neutralization efficiency of the hCypA and neutralizing antibody using in vitro enzyme‐linked immunosorbent assay (ELISA) (Figure S3E). hCypA interacts with RBD and interferes with the binding of the ACE2–RBD complex in blood samples. The MILF strip assay with standard blood samples (IgG and neutralizing antibodies containing negative blood) was used (Figure 5a). All blood samples were validated using the SD Biosensor SARS‐CoV‐2 Antigen Self‐Test Kit. For the neutralizing antibody containing blood sample, the band disappeared on the T line, but in SARS‐CoV‐2 IgG containing blood, the band appeared at both the T and R lines. To confirm the hCypA‐neutralizing effect in blood samples, negative blood samples were spiked with hCypA. The T line band disappeared as the hCypA concentration increased in the MILF strip assay (Figure 5b). The band intensity of the T line significantly decreased with an increase in the hCypA concentration (Figure 5c). These results showed that hCypA bound to RBD and interfered with the binding of RBD and ACE2. The absence of a band at the test line suggested that hCypA has neutralizing ability against SARS‐CoV‐2 RBD, which can prevent SARS‐CoV‐2 from infecting the host cell. Our MILF strip assay is rapid, functional, robust and can be used for the timely detection and diagnosis of SARS‐CoV‐2 variants.

## 3 DISCUSSION

The COVID‐19 pandemic and its rapid spread worldwide have triggered a global health emergency. Numerous therapeutic approaches have been proposed, and the recognition of cell receptors by CoVs is critical for the determination of viral infection, pathogenesis, and host range. The SARS‐CoV‐2S protein is the main protein used as a target in COVID‐19 vaccines. The RBD of the S protein binds to the ACE2 receptor on host cells and initiates virus‐host cell membrane fusion, which is crucial for viral infection. As a result, screening inhibitors that inhibit RBD–ACE2 interaction are critical for the treatment of COVID‐19. One of the most effective strategies to inhibit viral entry is targeting host or virus‐related components by directly blocking or indirectly interfering with the interaction between RBD and human ACE2. The RBD, as the key region for binding receptors, attracts antibodies and proteins targeting the conserved residues and offers great potential for the development of potent cross‐reactive therapeutic agents against various CoVs. The binding affinity described in this study showed that ACE2 bound marginally strongly to the SARS‐CoV‐2S protein relative to SARS‐CoV, as they have high structural similarity, coinciding with similar findings reported by other groups.^27^, ^33^, ^40^

SARS‐CoV‐2 binding to ACE2 is dominated by the RBD/ACE2 interface and blocking the interacting residues of RBD can inhibit specific binding to the ACE2 receptor. The protein hCypA plays a critical role in the life cycle of many CoVs (HCoV‐229 E, HCoV‐NL63, FPIV, SARS‐CoV, and MERS‐CoV).

In our study, protein–protein interactions using bioinformatics tools indicated that hCypA binds to SARS‐CoV‐2 RBD and that SPR, far‐western blot, and MILF strip assays revealed their binding interactions. Protein structure prediction and protein–protein interaction analysis using bioinformatics tools can help screen new drugs against other variants or diseases. We observed that extracellular hCypA binds SARS‐CoV‐2 RBD at the key RBM residue interface involved in interactions with the ACE2 receptor and overlaps with the ACE2 binding region. This hinders virus interactions, thereby mitigating its activity and restricting the entry of SARS‐CoV‐2 into the host cell. A strong interaction between the hCypA–RBD complex, as demonstrated by SPR and structural analysis, decreased the RBD‐ACE2 binding capacity, working as a masking mechanism to reduce RBD exposure to the ACE2 receptor. The hCypA acts as a potential inhibitor that can efficiently block SARS‐CoV‐2 binding.

The CsA molecule can be used as an immune‐suppressor to inhibit the binding of hCypA to the SARS‐CoV‐2 RBD and to control the hCypA mechanism. In addition, the well‐known CsA molecule inhibits the replication of various viruses by binding to intracellular human cyclophilins, which bind to the SARS‐CoV nucleocapsid protein.^16^, ^41^ Although hCypA can inhibit SARS‐CoV‐2 binding to host cells, even if the virus penetrates into the cell, it can interrupt viral replication by inhibiting the nucleocapsid–hCypA complex through CsA in the latent stage of viral infection.^42^, ^43^ The emergence of variants with mutations in the S protein affects the binding ability of the virus to the ACE2 receptor and exhibits high transmissibility and faster spreading. SPR results showed that the variants bind tightly to hCypA, suggesting no significant alterations to the complex structure due to mutations in the residues in the variants. The hCypA protein overlaps the RBM on all the variants and prevents the protein from tightly binding to the ACE2 receptor, except for the delta variant. Therefore, the delta variant evades the neutralizing effect of hCypA, as the T478K mutation on the RBD causes steric hindrance due to the surface potential shift from neutral to positive, which reduces stability and forces the flexible loop region on hCypA to shift away from the RBM region. The ACE2 receptor easily binds to the hCypA–delta RBD complex in open RBM regions, resulting in swift virus entry into the host cell. The N501Y mutation on RBD increases binding to ACE2 receptor; however, the combination of Q498R with N501Y in the omicron variant is suspected to further increase the binding affinity with ACE2. The strong binding of hCypA on the mutated RBD residue interface on omicron variant is expected to not only prevent its spread but also neutralize its transmissibility. The hCypA–RBD interaction highlights a new strategy for preventing a possible SARS‐CoV‐2 infection pathway against host cells and serves as a feasible approach for preventing SARS‐CoV‐2 infection. The MILF strip assay results also confirmed the binding mechanism of hCypA with RBD and its variants, where hCypA inhibits the binding of variants to the ACE2 receptor, except for the delta variant. The hCypA protein binds to the RBD of SARS‐CoV‐2 with a high affinity and possesses neutralizing ability. We visually confirmed the protein–protein binding interactions using the MILF strip assay. It can be used as a tool for evaluating the protein–protein interactions and molecular binding forces. Furthermore, MILF strip can be used to determine the inhibitory effect of hCypA on SARS‐CoV‐2 RBD and its variants.

## 4 MATERIALS AND METHODS

### 4.1 Protein and buffers

The SARS‐CoV‐2 variants of concern, that is, Alpha (United Kingdom, B.1.1.7: N501Y), Beta (South Africa, B.1.351: K417N, E484K, N501Y), Gamma (Japan/Brazil, P.1: K417T, E484K, N501Y), and Delta (India, B.1.617.2: L452R, T478K)), Omicron (South Africa BA.1(B.1.1.529): G339D, S371L, S373P, S375F, K417N, N440K, G446S, S477N, T478K, E484A, Q493R, G496S, Q498R, N501Y, Y505H), Epsilon (US‐California, B.1.427: L452R), Kappa (India, B.1.617.1: L452R, E484Q), Lambda (Peru, C.37; L452R, F490S), Deltacron (AY.4/BA.1: G339D, S371L, S373P, S375F, K417N, N440K, G446S, L452R, S477N, T478K, E484A, Q493R, G496S, Q498R, N501Y, Y505H), and Anti‐SARS‐CoV‐2 Neutralizing Antibody were purchased by Sinobiological (China, Beijing). Recombinant ACE2 and human Cyclophilin A were purchased from Thermo Fisher Scientific (WALTHAM, MA, USA). Anti‐Human IgG antibody and Anti‐rabbit IgG were purchased by sigma (St. Louis, MO, USA). The MILF buffers were used: 2.8 mM Triglyceride, 1 mM Ascorbic acid, 55 mM Hemoglobin, and 3 mM Bilirubin.

### 4.2 Far‐western blotting

We used the Yuliang Wu far‐western blotting method^35^; 20 μg of all purified proteins (RBD, hCypA, and ACE2) was loaded into wells with 62.5 mM Tris–HCl pH 6.8, 10% glycerol, 1% SDS, 1% β‐mercaptoethanol and 0.01% bromophenol blue for 5 min at 95°C. Total proteins were separated using 4%–12% SDS‐PAGE at 120 mA for 2 h and transferred to a Polyvinylidene Fluoride (PVDF) membrane (Amersham, USA) at 100 V for 2 h. The membrane was stained with Coomassie Brilliant Blue and Ponceau S (Sigma, USA) to determine whether the proteins had transferred from the gel to the membrane. Next, the proteins were denatured and refolded on the membrane in the AC buffer (100 mM NaCl, 20 mM Tris–HCl pH 7.6, 0.5 mM EDTA, 10% glycerol, 0.1% Tween‐20, 2% skim milk, and 1 mM DTT) by gradually reducing the guanidine–HCl concentration. The membrane was then blocked with 5% (w/v) blocking agent (GE Healthcare, USA) for 1 h at RT and incubated with 10 μg purified “bait” RBD protein RBD in PBS overnight at 4°C. The membranes were washed five times with PBST buffer (PBS containing 0.1% [v/v] Tween 20) and incubated with anti‐RBD antibody for 2 h at 4 °C. After incubation, the membrane was washed with PBST buffer three times and probed with an anti‐rabbit secondary antibody (Sigma, USA) for 1 h at RT. Immunoreactive proteins were detected using a WesternBright ECL detection kit (Advansta, USA).

### 4.3 Surface plasmon resonance

The binding affinity of hCypA to RBD proteins and variants was analyzed by SPR using a Biacore X‐100 instrument at 25°C. HBS‐EP buffer (10 mM HEPES pH 7.4, 150 mM NaCl, 3 mM EDTA, 0.005% polysorbate 20 v/v) was used as the running buffer at a flow rate of 10 μl/min, and flow cell 1 was used as a reference. To immobilize the hCypA protein to the CM5 sensor chip, the Au surface of the sensor chip was pretreated with HBS‐EP buffer and activated with a 1:1 mixture of 0.05 M N‐hydroxysuccinimide (NHS) and 0.2 M *N*‐ethyl‐*N*′‐(dimethylaminopropyl) carbodiimide (EDC) by modifying the carboxymethyl groups of dextran. The hCypA protein was diluted in 10 mM sodium acetate (pH 4.0) and then injected over the sensor surface to coat the surface of flow cell 2, followed by injection of 1 M ethanolamine hydrochloride (pH 8.5) to block the remaining active sites. After baseline stabilization, RBD proteins (all wild‐type RBD proteins) at different concentrations (0, 10, 50, 100, and 200 nM) were injected over flow cells 1 and 2 for 180 s at a flow rate of 10 μl/min with a 360 s dissociation time. Following each experiment, the sensor chip was regenerated with 10 mM glycine pH 2.5. The SPR analysis and dissociation constants (*K*_*D*_) of the complexes were determined using the BIAevaluation software (Biacore, Sweden). The binding affinity between RBD and [hCypA + CsA] was also tested using RBD immobilized CM5 chip. The protein complexes, such as [RBD + hCypA] and [RBD + hCypA + CsA] and ACE2 binding affinity were then determined following the same procedure. ACE2 protein was immobilized on the CM5 chip and the complex [hCypA + RBD] injected into the chip at different concentrations (0, 10, 50, 100, and 200 nM).

### 4.4 Structural analysis

The protein structure of hCypA (Protein Data Bank ID: 3K0M)^44^ was downloaded, and energy minimization using the energy minimization module of MOE^45^ was carried out after removing water molecules to make the structure applicable for docking. The structure of SARS‐CoV‐2S protein RBD was retrieved from PDB (PDB: 6M0J),^27^ and the ACE2 receptor structure bound to the S protein was also obtained. These structures were then copied in separate PDB files and minimized to obtain the optimal structures for docking. The SARS‐CoV‐2S protein was first docked with hCypA to obtain SARS‐CoV‐2–hCypA complexes, which were further docked with ACE2 to study the impact of hCypA on S protein ACE2 interactions.

The protein–protein docking module of the MOE 2020.0901 (Chemical Computing Group, Canada) was used for docking all cases. The AMBER10 force field function was used to calculate the binding energies, and the docking parameters were set as default (rigid body docking). The binding energies and root‐mean‐square deviation of atomic positions (RMSD) values of the docked complexes were analyzed to select the final complexes. The structure files were visualized and analyzed using the MOE and Pymol software.

### 4.5 MILF strip assay

A MILF strip assay was constructed with four constructs: a sample pad, conjugation pad, nitrocellulose membrane, and absorption pad. Figure S3A shows a schematic diagram of the MILF strip assay. To detect neutralizing antibodies in the blood or serum, AuNPs were conjugated to RBDs. The gold nanoparticles were conjugated to rabbit IgG as a control. The reaction between the AuNPs and the RBDs (or antibodies) was affected by the pH, and effective pH values were identified. First, solutions containing AuNPs were adjusted to a pH of 8.4. Figure 4a shows an illustration of the immunochromatographic test results. The performance of the MILF strip assay was affected by the treatment of the nitrocellulose membrane. Nitrocellulose membranes were blocked with PBS and dried at 37°C for 1 h. Human ACE2 protein (1 mg/ml) as the T line, 1 mg/ml anti‐IgG antibody as the R line, and 1 mg/ml anti‐rabbit IgG antibody as the C line were manually spotted on the nitrocellulose membrane. Conjugation pads were blocked with PBS containing 2% BSA and dried at 37°C for 4 h. Gold nanoparticle‐RBDs (or antibodies) were applied to the conjugation pad and dried at 37°C for 1 h. The sample pad was assembled on the conjugation pad with a 3 mm overlap. The conjugation and absorption pads were attached to both ends of the nitrocellulose membrane with a 3 mm overlap.

To examine the analytical performance of the lateral flow, we prepared artificial samples containing different concentrations of hCypA (0, 0.1, 1, 2, 5, 10, 20, 50, 100, and 200 μg in PBS) and 200 μg BSA, by mixing them with 0.05% Tween‐20. Each sample (30 μl) was loaded onto the sample pad of the prepared MILF strip assay and allowed to flow through the path for 15 min. The colorimetric signal generated by the reaction was captured using a cellular phone (iPhone 12pro, Apple, USA) and analyzed using ImageJ software (NIH, USA). The band intensity was converted to peak intensity using the software. The band intensity represents the calibration graphs, and the ratio of the T line to the C line band intensity (T/C ratio) was calculated. The T/C ratio was expressed as the sum of the band intensity values of the T and C lines. Blood samples with EDTA containing IgG and neutralizing antibodies and negative blood samples were purchased from RayBiotech (USA). Preprocessed blood samples were used for the performance analysis of lateral flow (Figures 5b and S3C,F).

## AUTHOR CONTRIBUTIONS

**Simranjeet Singh Sekhon:** Conceptualization (equal); data curation (equal); validation (equal); visualization (equal). **Woo‐Ri Shin:** Conceptualization (equal); formal analysis (equal); validation (equal); writing – original draft (equal). **Kim Sang Yong:** Methodology (equal); writing – review and editing (equal). **Jeong Dong‐Seok:** Data curation (equal); resources (equal); validation (equal); visualization (equal). **Choi Wooil:** Conceptualization (equal); data curation (equal); resources (equal); validation (equal); visualization (equal); writing – review and editing (equal). **Choi Bong‐Keun:** Formal analysis (equal); supervision (equal); writing – review and editing (equal). **Min Jiho:** Conceptualization (equal); formal analysis (equal); validation (equal); writing – review and editing (equal). **Kim Yang‐Hoon:** Conceptualization (equal); funding acquisition (equal); project administration (equal); resources (equal); writing – original draft (equal); writing – review and editing (equal).

## Supporting information


**Appendix S1.** Supporting Information.

## Data Availability

All data generated or analyzed for this study are available from the corresponding author upon reasonable request.
