# Supplementary material for: Cyclophilin A‐mediated mitigation of coronavirus SARS‐CoV‐2
Source: Bioeng Transl Med. 2022 Oct 27;8(2):e10436. doi: 10.1002/btm2.10436 (PMC9874840; doi:10.1002/btm2.10436)
Supplement: Supplementary file 1 — Appendix S1. Supporting Information. [file BTM2-8-e10436-s001.docx]

**[Supporting Information]**

**Cyclophilin A mediated mitigation of coronavirus SARS-CoV-2**

*Simranjeet Singh Sekhon^1^****^†^****, Woo-Ri Shin^1^****^†^****, Sang Yong Kim^2^****^†^****, Dong-Seok Jeong^3^, Wooil Choi^4^, Bong-Keun Choi^5^, Jiho Min^4*^, Ji-Young Ahn^1*^ and Yang-Hoon Kim^1*^*

^a^Department of Microbiology, Chungbuk National University, 1 Chungdae-Ro, Seowon-Gu, Cheongju 28644, South Korea

^b^Department of Food Science and Biotechnology, Shin Ansan University, 135 Sinansandaehak-Ro, Danwon-Gu, Ansan 15435, Republic of Korea

^c^1210, 76, Jikji-daero 436beon-gil, Heungdeok-gu, Cheongju-si, Chungcheongbuk-do, Republic of Korea

^d^Graduate School of Semiconductor and Chemical Engineering, Jeonbuk National University, Jeonju 54896, Korea, ^e^NUON Co., Ltd, Jungwon-gu, Seongnam, Gyunggi 13201, Korea

**^†^**These authors contributed equally to this work.

*Correspondence should be addressed to Yang-Hoon Kim ([kyh@chungbuk.ac.kr](mailto:kyh@chungbuk.ac.kr)), Ji-Young Ahn ([jyahn@chungbuk.ac.kr](mailto:jyahn@chungbuk.ac.kr)), and Jiho Min ([jihomin@jbnu.ac.kr](mailto:jihomin@jbnu.ac.kr))

Keywords: cyclophilin; coronavirus; spike protein receptor binding domain; surface plasmon resonance, COVID-19 variants

[Contents]

Figures

1. Supplementary Fig. S1
2. Supplementary Fig. S2
3. Supplementary Fig. S3

**Supplementary Fig. S1**

**Fig. S1.**

(A) Structural alignment of the RBDs of SARS-CoV-2 (orange) and SARS-CoV (olive). Contacting residues in the SARS-CoV-2 RBD and SARS-CoV RBD are indicated including K417/V404, L455/Y442, F456/L433, A475/P462, F486/L472, Q493/N479, Q498/Y484, N501/T433, V503/I489 of SARS-CoV-2/SARS-CoV. (B) Structural representation of the S protein RBD (olive) complexed with SARS-CoV receptor ACE2 (light blue). (C) Structural alignment of the RBDs of SARS-CoV-2 (orange) and SARS-CoV (olive) and their binding modes to the ACE2 receptor (light blue). (D) Structural representation of the CsA (deep blue) complexed with hCypA (pink). The key residues that take part in the interaction are also shown. (E) The SPR binding affinity of RBD binding to ACE2 (a), hCypA binding to ACE2 (b), and CsA binding to hCypA (c) is shown. The CsA is not binding to ACE2 (d) and RBD (e). (F) Structural representation of hCypA (pink) and S protein RBD of SARS-CoV (olive) docked complex with interacting region residues labeled on the complex structure. (G) SPR affinity analysis of the hCypA-SARS-CoV RBD.

Figure 1E shows the binding properties among the protein complexes, [RBD]-[hCypA], [RBD]-[hCypA+CsA], [ACE2]-[RBD+hCypA], and [ACE2]-[RBD+hCypA+CsA]. The RBD binds to hCypA with a binding affinity K_D_ value of 6.85 × 10^-8^ M (Fig. 1Ea). The hCypA binds to CsA with a binding affinity *K_D_* value of 2.07 × 10^-10^ M (Fig. 1C and Fig. S1Ec). Moreover, CsA molecule binds to hCypAs' RBD binding region (Fig. 1C). Therefore, it is believed that the [RBD]-[hCypA] interaction could be effectively inhibited by CsA. In other words, the hCypA binds to CsA more tightly than RBD, that can cause the low binding value, 1.84 × 10^-5^ M, with RBD.

Besides, our structural analysis demonstrated that the hCypA can bind to RBD at the ACE2 binding sites. As shown in Fig. 1A, the most amino acid residues involved in the binding interface between RBD and hCypA are located in correspondence of the RBD-ACE2 interface. In order to validate, we first manufactured the [RBD+hCypA] complex, and reacted it with ACE2. The RBD binds to ACE2 with a binding affinity KD value of 4.48 × 10^-8^ M (Fig. S1Ea). However, the interaction between [ACE2] and [RBD+hCypA] showed lower binding affinity *K_D_* value of 1.60 × 10^-5^ M (Fig. 1Ec). Therefore, the hCypA can inhibit the binding between RBD and ACE2. We additionally manufactured the protein complexes including RBD, hCypA and CsA, and the complexes were then reacted to ACE2 protein. The binding affinity value of 8.58 × 10^-7^ M (Fig. 1Ed) can be explained that the interaction between hCypA and RBD was blocked by CsA, and the uncomplexed RBD bound to ACE2 protein.

**Supplementary Fig. S2**


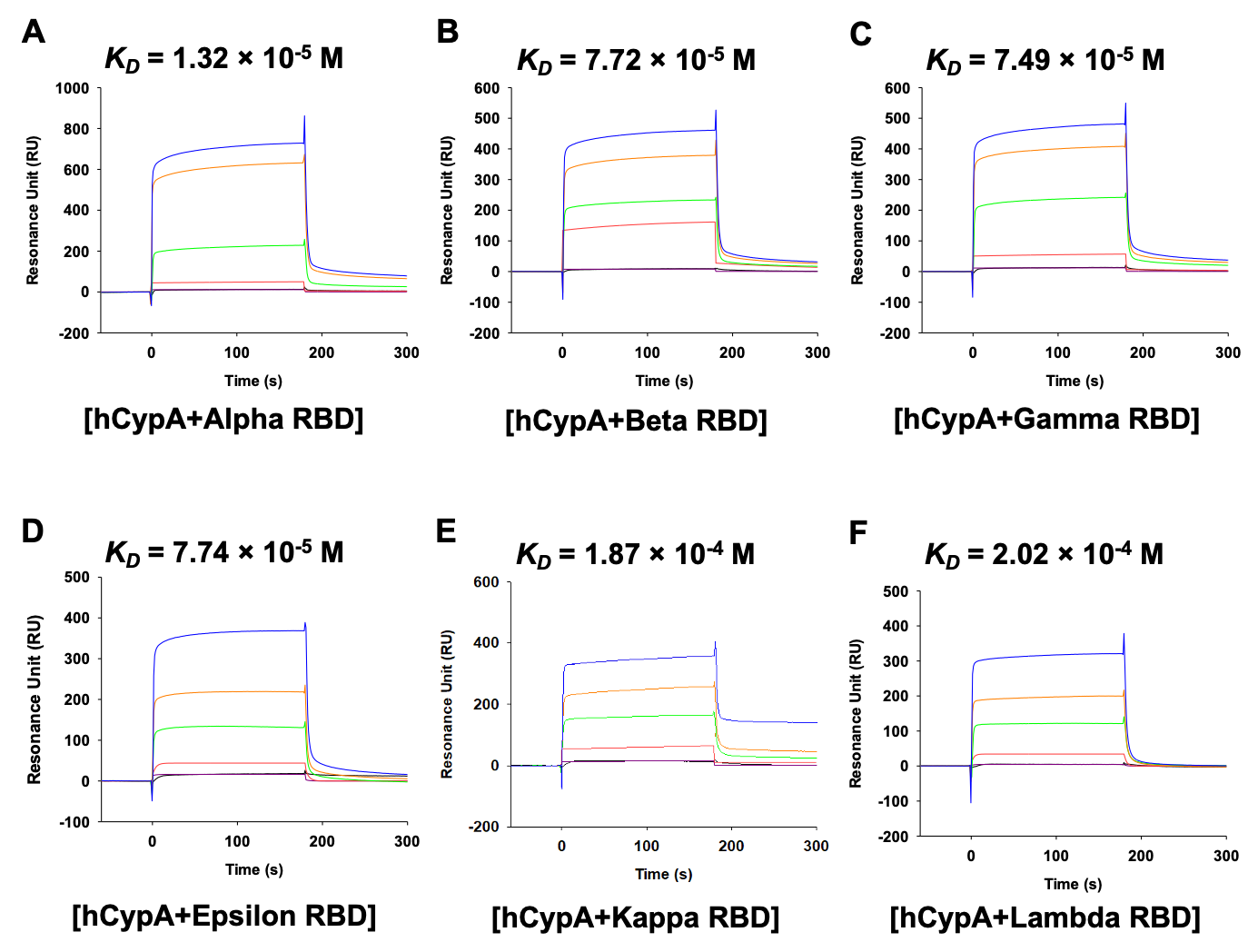


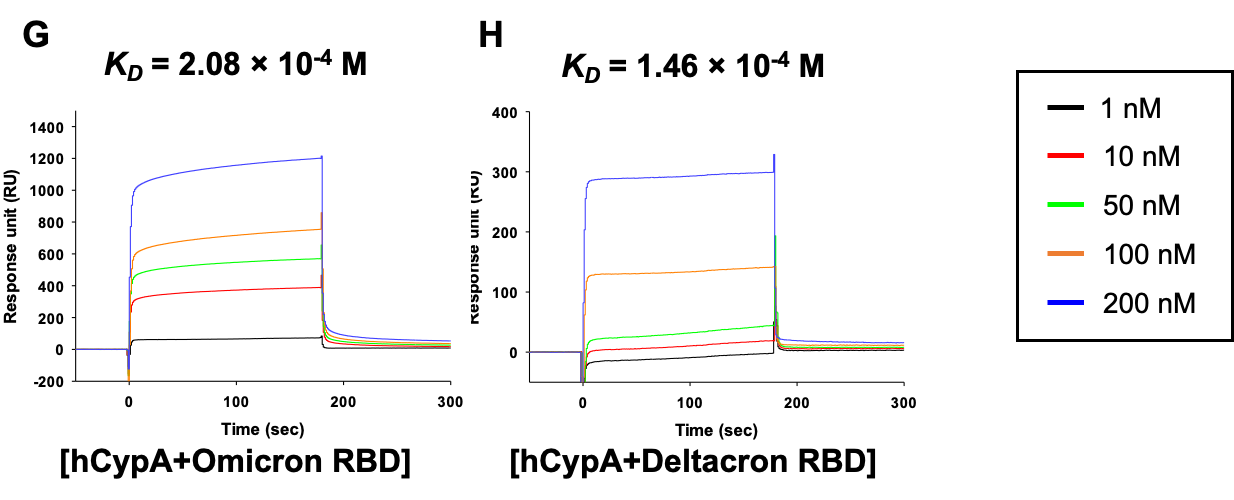


**Fig. S2**. SPR affinity analysis of the ACE2 with [hCypA+RBD variants complex]. (A) [ACE2]-[hCypA+Alpha RBD], (B) [ACE2]-[hCypA+Beta RBD], (C) [ACE2]-[hCypA+Gamma RBD], (D) [ACE2]-[hCypA+Epsilon RBD], (E) [ACE2]-[hCypA+Kappa RBD], (F) [ACE2]-[hCypA+Lambda RBD], (G) [ACE2]-[hCypA+Omicron RBD], and (H) [ACE2]-[hCypA+Deltacron RBD]. The *K_D_* value of binding affinity is shown in Table 3.

**Supplementary Fig. S3**


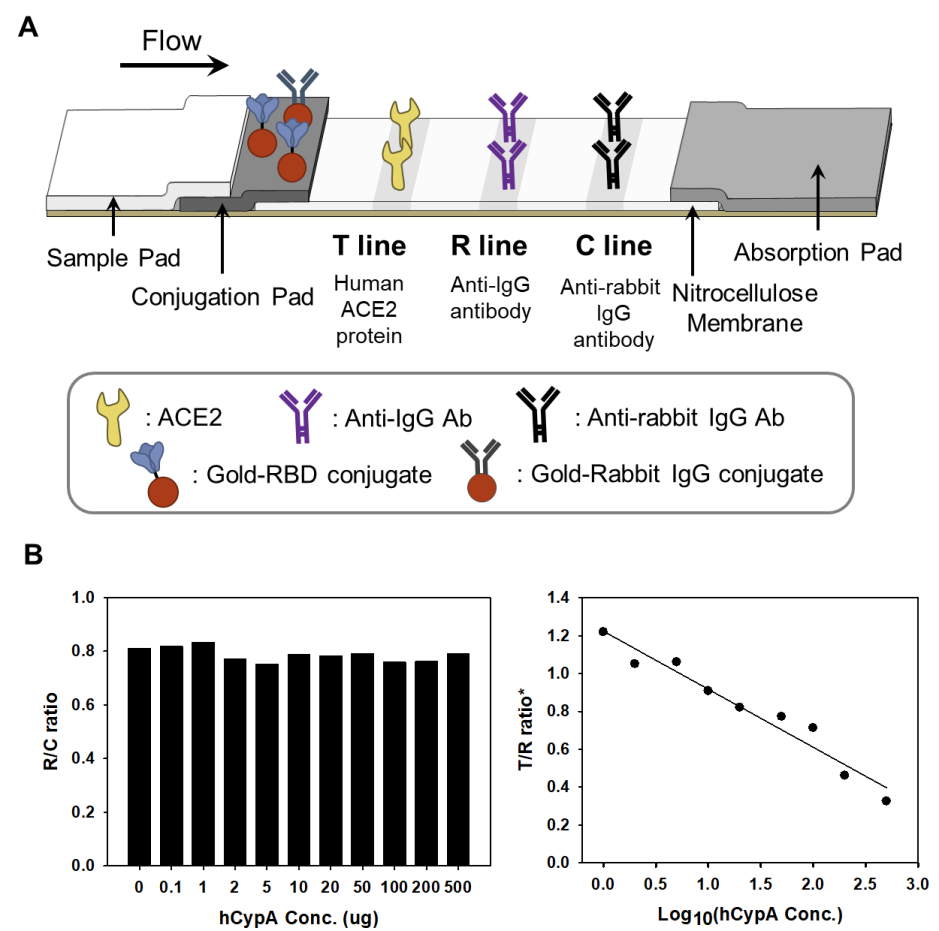


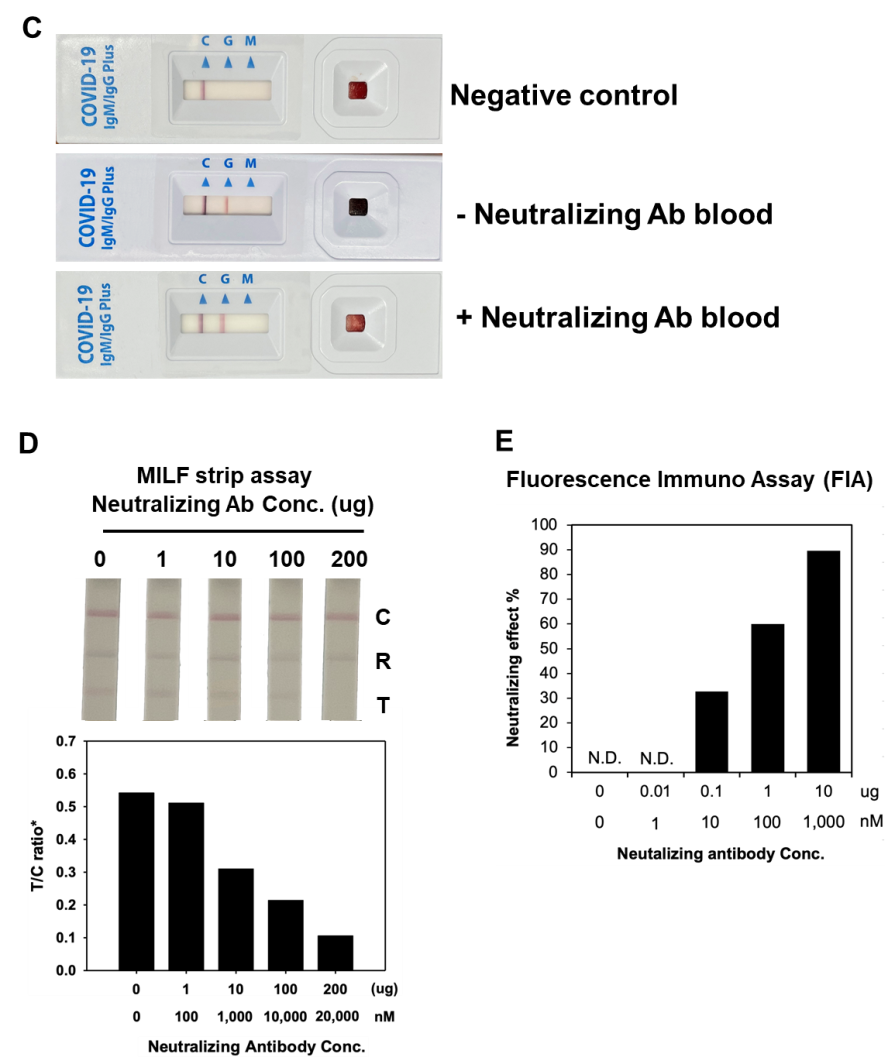


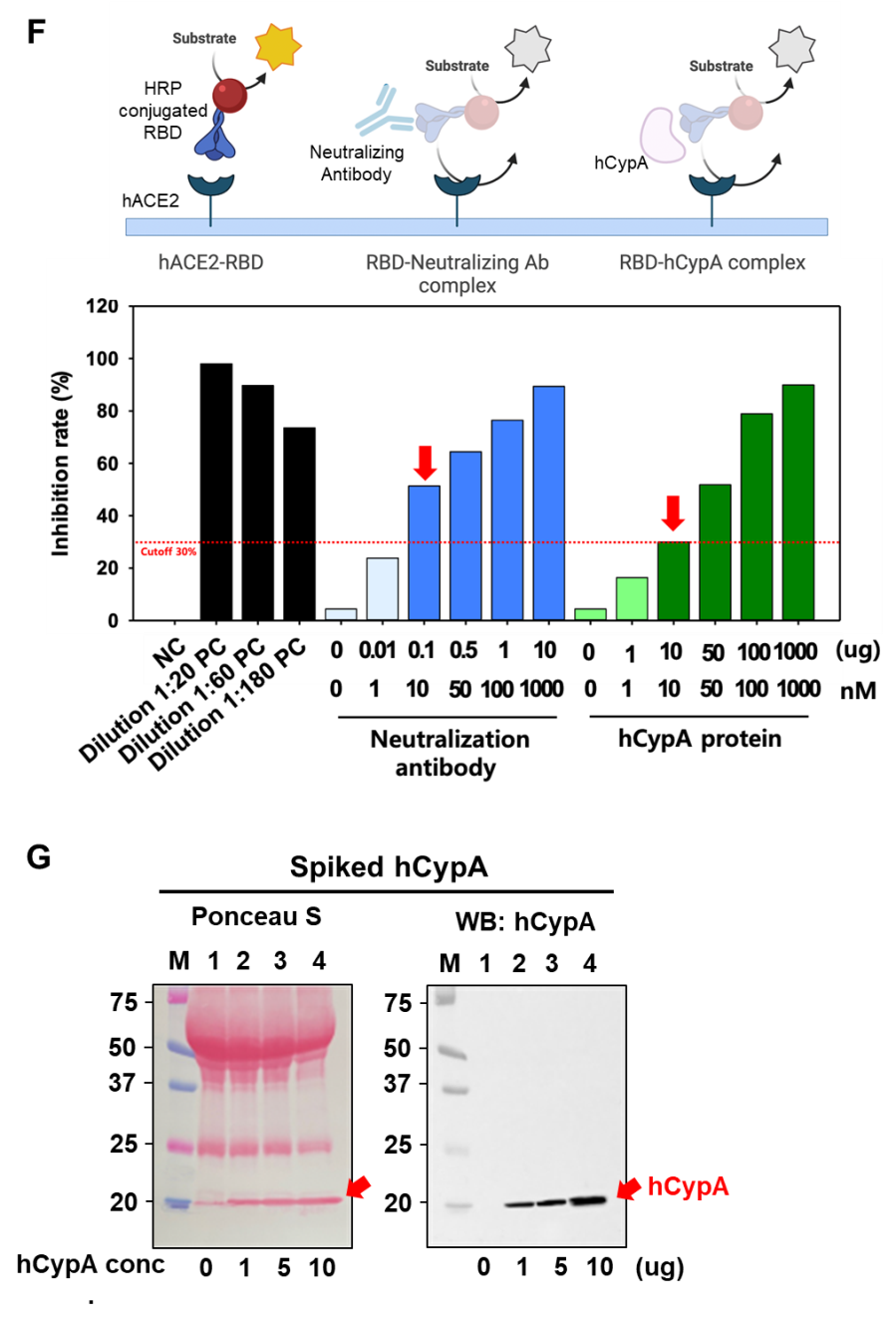


**Fig. S3.** (A) Illustration of the MILF strip assay for neutralizing antibody test to SARS-CoV-2. (B) The calibration graph as control line (C line), test line (T line) and reaction line (R line) ratio of hCypA concentration. R/T ratio (left) and T/R ratio (right). (C) Image of the STANDARD Q COVID-19 IgM/IgG Plus Test (SD biosensor, Korea) for the detection of neutralizing antibodies to SARS-CoV-2 present in human whole blood, - neutralizing antibody IgG blood represent the blood sample without neutralizing antibody, + neutralizing antibody IgG blood represent the blood sample with neutralizing antibody. (D) MILF strip assay. Binding interference between ACE2 and SARS-CoV-2 RBD was tested using neutralizing antibody. The calibration graph as control line (C line) and test line (T line, ACE2 zone) ratio of neutralizing antibody concentration. (E) Fluorescent Immuno Assay (FIA). The inhibition rate (%) of neutralizing antibody was obtained by using AFIAS COVID-19 nAb kit (bodietch, Korea). (F) Results of SARS-CoV-2 neutralizing antibody detection ELISA assay (Genscript) for extracellular neutralization efficiency using different concentration of hCypA and neutralizing antibody at SARS-CoV-2 RBD. Schematic image was illustrated by BioRender.com. Red dotted line indicates the positive inhibition rate guideline, value at 30%. Red arrows show the neutralization value of neutralizing antibody and hCypA protein, 51% and 30% respectively. (G) Image of the spiked hCypA protein in normal human serum by western blotting analysis

We compared the inhibition efficiency between the hCypA and SARS-CoV-2 neutralizing antibody. First of all, we tested the MILF strip assay with dilutions of the SARS-CoV-2 neutralizing antibody (0-200 μg). As shown in Fig. S3D, it shows images of the MILF strip bands exposed to samples containing different concentrations of neutralizing antibodies. The band intensity disappears at the T line (ACE2 zone) as the antibody concentration increases, the T line band disappears at the 200 μg of neutralizing antibodies. The T/C ratio of neutralizing antibody is decreased with neutralizing antibody concentration increases.

Also, we compared the neutralization efficiency of the hCypA and neutralizing antibody using SARS-CoV-2 neutralizing antibody detection ELISA assay (Genscript Cat#. L00847-A). The spiked therapeutic Mab (see a part of neutralizing Ab in graph, ranging from 0 to 10 ug) was used and the results were compared with hCypA (ranging from 0 to 1000 ug). As shown in Supplementary Figure S3F, we confirmed the SARS-CoV-2 neutralizing effect of hCypA protein. To assure the validity of the results, the neutralization activity was analyzed by comparing the inhibition rate (%) to the following.

Inhibition rate = (1 - OD value of Sample / OD value of Negative Control) × 100%

The cutoff value is analyzed that the positive (SARS-CoV-2 neutralizing effect detected) is upper 30% inhibition rate in this kit. hCypA (10 nM, 10 ug) was shown ~30% inhibition rate (see red-dotted line and arrow), which was contained within the value at around 1 nM ~10 nM of neutralizing Ab.
